# Editorial—Global Climate Change and Contaminants

**DOI:** 10.3390/ijerph120707582

**Published:** 2015-07-07

**Authors:** Hans Sanderson, Michael Goodsite

**Affiliations:** 1 Department of Environmental Science Section of Environmental Chemistry and Toxicology Aarhus University Frederiksborgvej 399, DK-4000 Roskilde, Denmark; 2 Department of Technology and Innovation University of Southern Denmark Niels Bohrs Allé 1, DK-5230 Odense M, Denmark; E-Mail: hasa@dmu.dk

This Special Issue in the *International Journal of Environmental Research and Public Health* focuses on the inter-linkage between the global distribution of contaminants and climate change. The climate has always changed, but now the international scientific community expects that anthropogenic-induced climatic change will lead many places to experience weather and environments that are warmer and wetter than expected, with more extreme weather incidents occurring. Since global contaminants are also affected by environmental and climatic factors, the changing climate has and will undoubtedly continue to affect global processes surrounding the release, volatilization or otherwise; transport; chemical or physical conversion in the atmosphere or other media; deposition; and environmental partitioning of contaminants that already have, or may potentially have, spread across the world with health consequences that may follow. This issue will address the problems associated with climate change and global contaminants, including those related to adaptation, impact, vulnerability of the natural and human environment, human health assessments and regulatory policies. Adaptation to climate change and the implicit policy and economic assessments hereof will also be addressed.

In the context of public health, in this issue, global climate change has at least three major impact routes:

1. Acute impacts/disasters as a consequence of extreme weather e.g., storms such as Haiyan and Sandy.
2. Gradual increases of direct climate risk factors across the globe, e.g., drought, heat stress, and wild fires, not related to singular extreme events.
3. Indirect climate change enhanced risk factors, e.g., disease vectors and zoonosis, subtle mental effects [1], and the distribution and thus human exposure of atmospheric contaminants.

All three routes are being considered in relation to disaster risk reduction and adaptation to climate change in different parts of the world with climate change risk assessments. It is already known that the poorest people are being/will be the hardest hit by the public health impacts of climate change (WHO, 2014 [2]). Sensitive sub-populations are of specific concern: elderly, children, and patients, e.g., with respiratory diseases. The recent WHO forecasting report concludes that climate change is expected to cause approximately 250,000 additional deaths per year between 2030 and 2050; 38,000 due to heat exposure in elderly people, 48,000 due to diarrhea, 60,000 due to malaria, and 95,000 due to childhood under- and malnutrition. Results indicate that the burden of disease from climate change in the future will continue to fall mainly on children in developing countries, but that other population groups will be increasingly affected (WHO, 2014 [2]).

In terms of risks related to atmospheric contaminants, e.g., ground-level ozone, particulate matter (PM_x_), SO_2_, NO_x_, and pollen/allergens, it is important to factor in the global mega-trend of increasing urbanization. It is estimated that half of the world’s population today live in ever larger and expanding cities—and that 1.5 million more people every week move to large cities globally (PWC, 2014 [3]). Hence, poor air quality due to local production of contaminants becomes a potentially significant stressor on public health causing respiratory and cardiovascular diseases to an increasing portion of the world’s population.

The fate and transport of toxic contaminants are affected by weather conditions (e.g., temperature, wind, and precipitation), and some chemicals are, moreover, particularly relevant, as they involve activation to reactive species by ultra-violet radiation in sunlight (photoactivated toxicity), for example polycyclic aromatic hydrocarbons (PAHs). Other compounds can be transformed due to temperature to exacerbate their toxicity by inducing toxic and carcinogenetic reactive oxygen species (ROS), e.g., methyl mercury (MeHg) [4]. Human exposure to natural toxicants may also increase via food and water, such carcinogenetic mycotoxins (e.g., aflatoxins, and other toxins produced by mold) residues in foods. Algal toxins, e.g., blue-green algae toxin microcystin-LR, which comprise heptatoxic, immonunotoxic and possibly carcinogenic compounds, are magnified in, e.g., fish and shellfish, caught typically from eutrophic harbor waters and shallow freshwater lakes; they are heat resistant and are therefore not removed during cooking. There is therefore a need for further elucidation of similar mechanisms and to incorporate these findings into the risk assessment of global contaminants [5].

While initiatives to adapt to the effects of climate change are growing in number, they may fail to achieve the desired outcomes unless critical competing interests are taken into account during the planning process. Four processes—enclosure, exclusion, encroachment and entrenchment—reflect the competing interests that occur in some adaptation initiatives and these can lead to inequitable outcomes [6]. Some policies such as those that make use of the *precautionary principle* may limit the effects of future releases of contaminants—but those already in the environment will continue to cycle in the environment and may continue to affect us.

This Special Issue is focused on global contaminants and climate change. It is our hope that the articles accepted into the issue provide the basis for a deeper understanding of the field, and as a basis for further work.

The research leading to these results has received funding from the European Community’s Seventh Framework Programme under Grant Agreement No. 308337 (Project BASE). The text reflects only the authors’s views and the EU is not liable for any use that may be made of the information contained therein.
